# Hydrogen-Bond Bifurcation as the Key Mechanism of Pyridine Surface Diffusion on MCM-41

**DOI:** 10.1021/acsomega.6c04133

**Published:** 2026-09-04

**Authors:** Ilya G. Shenderovich

**Affiliations:** Faculty of Chemistry and Pharmacy, University of Regensburg, Universitätsstr. 31, 93040 Regensburg, Germany

## Abstract

Loss of linearity significantly weakens hydrogen bonds. However, this energy penalty can be partially compensated by the formation of a second hydrogen bond. Theoretical calculations presented in this work demonstrate that such bifurcated hydrogen bonds play a key role in the surface diffusion of adsorbed molecules. In particular, for a model MCM-41 silica surface with characteristics consistent with experimental observations, the activation barrier for pyridine diffusion via the bifurcated hydrogen-bond mechanism is approximately 20 kJ/mol, which is significantly lower than the desorption energy of pyridine from the silica surface (>50 kJ/mol). Owing to the low density of surface silanol groups, MCM-41 approximates a limiting case in which the diffusion of pyridine on the silica surface requires overcoming an exceptionally high energy barrier. For most amorphous silica materials, the density of surface silanol groups is higher, and surface diffusion is therefore expected to dominate completely over desorption at room temperature when the surface coverage of pyridine is significantly below monolayer saturation.

## Graphical abstract

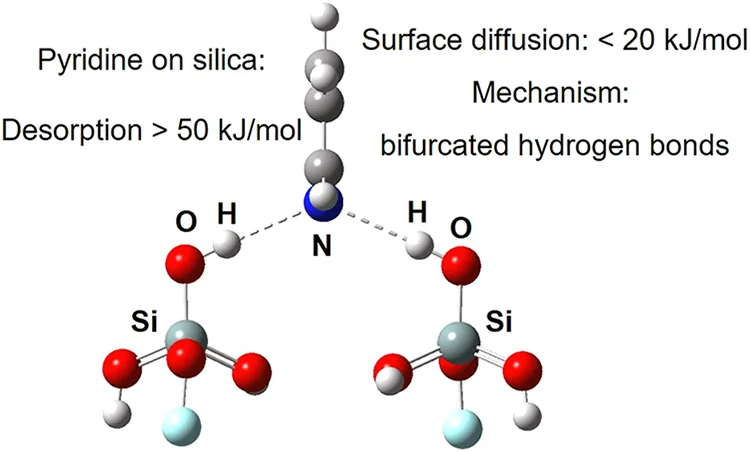

## Introduction

Mesoporous silica of the MCM-41 type represents a distinctive class of materials possessing amorphous silica walls but exhibiting a highly ordered hexagonal pore structure.^1,2^ Their pore diameter is uniform and can be systematically tuned between 2 and 4 nm, while the accessible internal surface area typically exceeds 1000 m^2^/g. These characteristics make MCM-41 materials suitable for size-selective catalytic applications.^3,4^

In the absence of specific functionalization, the chemical reactivity of the internal surface is governed by silanol groups. These groups also act as adsorption sites for guest molecules. Their effects on the crystallization,^5–7^ melting point,^8–10^ and dynamics of guest molecules have been examined in numerous studies.^11–13^ It is reasonable to expect that the spatial distribution of surface silanol groups determines whether the surface diffusion of adsorbed species resembles sliding along the surface or occurs via random jumps between neighboring, or even distant, silanol groups. If the first scenario is realized, the adsorbed molecule should be able to form bifurcated hydrogen bonds with surface groups, allowing it to slide along the surface without completely breaking its interaction with the surface.^14–17^ For example, even weak, nonlinear hydrogen bonds have been shown to play a decisive role in directing molecular self-assembly.^18–20^ In the second scenario, the adsorbed molecule would leave the surface, become temporarily unbound, and resemble a molecule in the gas phase before finding another surface-active group with which to interact.

Surface diffusion mechanisms are critical for understanding the properties of any adsorbent. However, it is often challenging to find examples in which the distribution of surface adsorption sites can be accurately determined or at least reliably assumed. In this context, MCM-41 represents a promising model system, since the well-defined ordering of its pores suggests that the surface structure of the material may also exhibit regular patterns. The validity of this assumption is supported by experimental data. Specifically, the surface of high-quality MCM-41 samples contains only silanol groups with uniform proton-donating ability, and when the coverage of adsorbed pyridine is significantly below a monolayer, the molecules migrate across the surface without desorption.^21,22^ Experimental studies of the molecular dynamics of pyridine adsorbed on a silica surface indicate that pyridine diffusion is not observed at temperatures of 221 K and below, but becomes apparent at 243 K.^23^

The first aim of this work is to construct the simplest possible pattern representing the distribution of silanol groups on the surface of an idealized MCM-41 sample. This pattern should correspond to the available experimental data. The primary objective is to examine whether the surface diffusion of a pyridine molecule on such a surface may occur according to the first scenario described above. Note that the scope of this work is limited to these two objectives and does not include the construction of a fully stable structure of the idealized MCM-41 sample that satisfies all surface property constraints.

## Computational Methods

The Gaussian 09.D.01 program package^24^ was used for geometry optimizations and NMR calculations. The ωB97XD^25^ DFT hybrid functional and the def2tzvp^26^ and basis sets were used. The geometry optimization was done at the Tight convergence criteria. NMR calculations were carried out using the GIAO approach. All calculations were done using the Polarizable Continuum Model (PCM) approximation with water as the solvent.^27,28^ This choice is arbitrary. The outcomes of the PCM approximation are not very sensitive to the value of the dielectric constant.^29^ However, this correction is necessary to at least approximately account for the effect of the environment.

One may argue that the gas-phase approximation is more appropriate than the PCM approximation when considering an adsorbed state. It is difficult to formulate a rigorously substantiated objection to this argument. However, the primary aim of the calculations reported here is to estimate the difference between the activation barrier for pyridine diffusion via the bifurcated hydrogen-bond mechanism and the desorption energy of pyridine from the silica surface. The use of the PCM approximation not only lowers both energies relative to the corresponding gas-phase values but, more importantly, reduces the difference between them.^30–33^ (This trend may change in systems involving significant charge transfer, but that is not the case here.^30,31^ ) Therefore, PCM calculations employing a high dielectric constant provide a conservative estimate and thus a lower bound for the difference between the activation barrier and the desorption energy.

It should be noted that for most reported structures, the positions of selected atoms were fixed and not optimized. Details for each structure are provided in the text. This approach, of course, introduces an error in the estimated energies of these structures. However, first, this error is small compared to the energy associated with the hydrogen bonds under discussion, and second, the calculated energy values are intended solely for qualitative rather than quantitative analysis. For the same reason, no correction for the basis set superposition error was applied.

## Results and Discussion

### Experimental Properties and Structural Models of the MCM-41 Surface

The structure of MCM-41 silica has been the subject of numerous experimental and theoretical studies. Perhaps the most reliable conclusion that can be drawn from these studies is that different MCM-41 samples exhibit significant variations in their structural properties. Some materials possess a non-negligible degree of microporosity.^34^ The diameter of these micropores is smaller than 0.7 nm.^35^

The number of surface silanol groups also varies considerably, ranging from about 3 OH/nm^2^ in experimental studies^21,36^ to about 7 OH/nm^2^ in theoretical models.^37–39^ Notably, the density of silanol groups on amorphous silica materials is about 5 OH/nm^2^ and appears largely independent of their origin and structural characteristics.^40^ The density of silanol groups can be significantly reduced upon dehydration and restored upon subsequent hydration.^40,41^ The silica surface may contain geminal silanol groups as well as hydrogen-bonded silanol groups.^42^

Pyridine molecules adsorbed on a silica surface form hydrogen bonds with silanol groups. Two types of such hydrogen bonds have been reported, with energies of approximately 52 and 91 kJ/mol.^36,43,44^ The former value was attributed to a complex of pyridine with two bridged hydroxyl groups, whereas the latter was assigned to pyridine bonded to a free hydroxyl group. Such an attribution appears surprising. The proton-donating ability of an isolated silanol group is insufficient to protonate pyridine. As a result, in their hydrogen-bonded complexes the O–H bond remains much shorter than the H···N bond. When the oxygen atom of a silanol group participates in hydrogen bonding with a neighboring silanol group, its proton-donating ability is enhanced. Consequently, the hydrogen bond formed between this silanol group and pyridine is expected to be stronger, i.e., to have a higher interaction energy. Therefore, the more reliable estimate for the hydrogen-bond energy between pyridine and an isolated silanol group is about 52 kJ/mol.

The main conclusion that follows from the discussion above is that none of the existing MCM-41 models is suitable for simulating in detail the adsorption of pyridine in an arbitrary MCM-41 sample. The analysis therefore has to be restricted to a particular sample or to a set of samples synthesized and prepared using strictly identical procedures. Accordingly, the model discussed below will be constructed so as to be in agreement with the experimental data reported for MCM-41 samples produced by Findenegg’s group.^21^

### Key Experimental Inputs for the Simplest MCM-41 Surface Model

The experimental data that the model must satisfy are as follows.^21^ The density of surface silanol groups is 3 OH/nm^2^. The amounts of geminal and bridging silanol groups, as well as of groups inaccessible to pyridine, are negligible. The proton-donating ability of these silanol groups is uniform. Their hydrogen bonding to pyridine results in a 25 ppm high-field shift of the ^15^N NMR signal. Based on an experimentally established correlation, such a shift corresponds to an H···N distance of approximately 1.67 Å.^21,45^ When the surface coverage of pyridine is below a monolayer, the molecules diffuse along the silica surface but do not desorb at room temperature. In high-quality MCM-41 samples, the surface roughness is sufficiently low that this diffusion does not lead to complete averaging of the anisotropy of the ^15^N NMR chemical-shift tensor. At the same time, the distance between neighboring surface silanol groups in these samples exceeds 0.5 nm.^46^

The largest possible distance between surface silanol groups at a density of 3 OH/nm^2^ is obtained when the groups are arranged in a hexagonal close-packed pattern. In this case, the distance between neighboring silanol groups is 6.2 Å. Formally, such an arrangement satisfies the above requirements. However, this packing imposes an unnecessarily strict restriction because it can be realized only in a single geometrical configuration. A square packing is also sufficiently dense, but in contrast to the hexagonal arrangement, it can be continuously deformed into various rhombic configurations. In this case, the distance between neighboring silanol groups is 5.77 Å. For simplicity, we therefore adopt a value of 5.7 Å. This value satisfies the requirements while providing reasonable flexibility in the distribution of the surface groups. Ultimately, the aim is not to determine the full range of possible activation barriers for pyridine surface diffusion but rather to estimate a realistic upper limit.

At the ωB97XD/def2tzvp level of theory, the hydrogen-bonded complex (HO)_3_Si–O–H···pyridine exhibits an H···N distance of 1.734 Å. This distance is slightly too long for our purposes. For the qualitative analysis that constitutes the main objective of this work, the only critically important factor is the proton-donating ability of the hydroxyl group interacting with pyridine. This parameter can therefore be adjusted to reproduce the experimental value by making minor modifications to the chemical nature of the remaining substituents. Such tuning may be achieved in various ways. In the present study, the specific choice was guided by the desire to preserve the [SiO_4_] structural motif while avoiding the use of excessively bulky substituents. When one of the hydrogen atoms is substituted by fluorine, forming (FO)­(HO)_2_Si–O–H···pyridine, the H···N distance contracts to 1.668 Å, which is very close to the target value of 1.67 Å. The calculated difference in the ^15^N NMR chemical shifts between pyridine in this complex and free pyridine is 27.6 ppm (GIAO, ωB97XD/def2tzvp). Considering the typical margin of error of ±2 ppm for such calculations,^47^ this result is in good agreement with the experimental value of 25 ppm.

### Model Surface Architecture

The strategy for constructing a model pattern of surface silanol groups is as follows. The geometry of the (FO)­(HO)_2_Si–O–H unit is taken from its complex with pyridine. Two such units, representing mirror images of each other, are used to form the desired pattern, with a Si···Si distance of 5.7 Å, denoted as Sil^in^ in Figure 1a. The positions of all atoms, except for those of the OH groups accessible to pyridine, are fixed and not optimized. Although this restriction slightly affects the energy of the molecular structures, the associated error is expected to be much smaller than the energy of the hydrogen bond to pyridine, which is the focus of this study. The silanol groups in this structure do not interact with each other, as shown by comparing its energy with that of Sil^out^, Figure 1b. The energy of Sil^in^ is 0.4 kJ/mol higher than that of Sil^out^.

**Figure 1:**
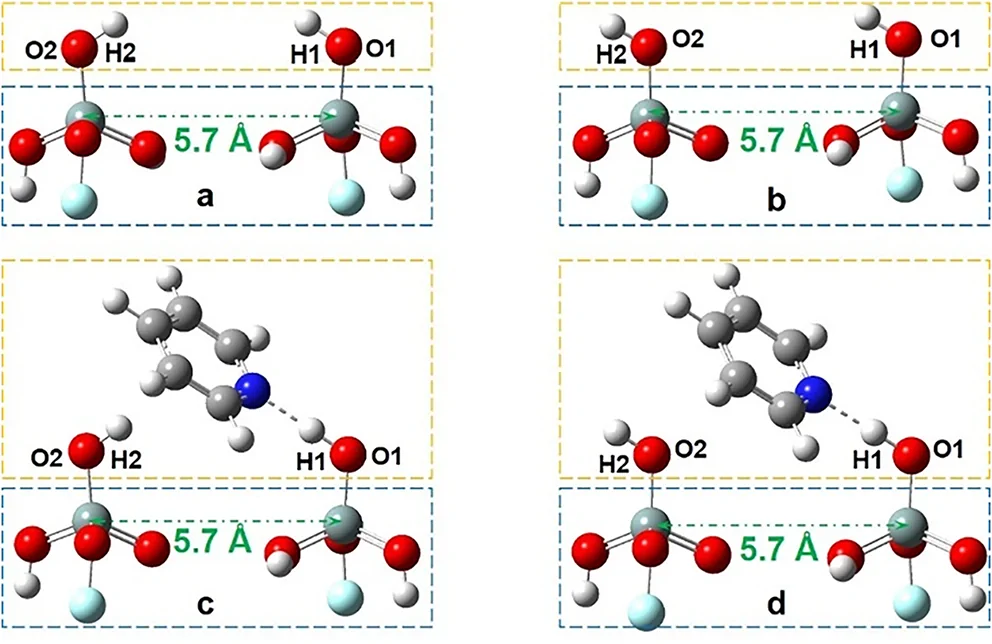
A model pattern of surface silanol groups of MCM-41 without and with adsorbed pyridine. The positions of the atoms in the blue region were fixed, whereas those in the orange region (H1, H2, O1, O2, pyridine) were optimized. Sil^in^ (a), Sil^out^ (b), Sil^in^_Py (c), Sil^out^_Py (d).

When a pyridine molecule interacts with the Sil^in^ structure, it forms a hydrogen bond with an H···N distance of 1.675 Å, Sil^in^_Py in Figure 1c and Table 1. The geometries of pyridine and the two upper hydroxyl groups (O1, H1, O2, H2) are optimized. The calculated difference in the ^15^N NMR chemical shifts between pyridine in this complex and free pyridine is 24.1 ppm. The energy of this complex is taken as 0 kJ/mol and is 52.8 kJ/mol lower than the sum of the energies of non-interacting Sil^in^ and pyridine in its optimized geometry, Table 1. This energy can naturally be assigned to the O1–H1···N hydrogen bond, a value that agrees with the estimation discussed above. To be precise, this energy includes a small contribution from the interaction between pyridine and the second hydroxyl group, O2–H2. This can be illustrated using the Sil^out^_Py structure, Figure 1d. The energy of Sil^out^_Py is 2.8 kJ/mol higher than that of Sil^in^_Py, Table 1. This difference comprises a positive contribution arising from the loss of interaction with the second hydroxyl group and a negative contribution associated with the strengthening of the primary hydrogen bond, as evidenced by the geometric changes reported in Table 1.

**Table 1:** Calculated Energies and Selected Geometric Parameters for the Studied Structures^a^

Structure	Energy (kJ/mol)	N···O1 (Å)	N···O2 (Å)	N···H1 (Å)	N···H2 (Å)
Sil^in^, pyridine	52.8	∞	∞	∞	∞
Sil^in^_Py	0.0	2.677	4.044	1.675	3.147
Sil^out^_Py	2.8	2.677	3.900	1.672	4.460
Sil^in^_Py_tr	19.9	3.172	3.172	2.204	2.204
Sil^out^_Py_tr	36.1	3.162	3.166	2.192	3.819
Sil^in^_Py_pretr	13.3	3.172	3.636	2.203	2.755
Sil^out^_Py_pretr	16.5	3.172	3.605	2.200	4.042

a Only O1, H1, O2, and H2 positions are optimized in all structures; further details are given in the text.

### Migration of Pyridine between Neighboring Silanol Groups

If pyridine can migrate between surface silanol groups without desorption, a transition state should exist in which its nitrogen atom forms two equivalent bifurcated hydrogen bonds with two neighboring silanol groups. To locate such a transition state, the following procedure was employed. The geometry of pyridine was taken from Sil^in^_Py. Pyridine was positioned relative to Sil^in^ such that its C_2_ symmetry axis lies in the Si–Si–O1–O2 plane, the aromatic ring plane is perpendicular to this plane, and the distances between the nitrogen atom and the two Si atoms are equal. These distances were varied gradually, and the positions of O1, H1, O2, and H2 were optimized at each step. The applied constraints inevitably affect the energies of the resulting structures. However, the associated errors can be neglected in this qualitative study.

The minimum energy was obtained for Sil^in^_Py_tr, Figure 2a. The energy of this transition state is 19.9 kJ/mol higher than that of Sil^in^_Py and 32.9 kJ/mol lower than the total energy of non-interacting Sil^in^ and pyridine, Table 1. Therefore, surface migration through bifurcated hydrogen bonds is considerably more energetically favorable than jumps involving complete hydrogen bond dissociation.

**Figure 2:**
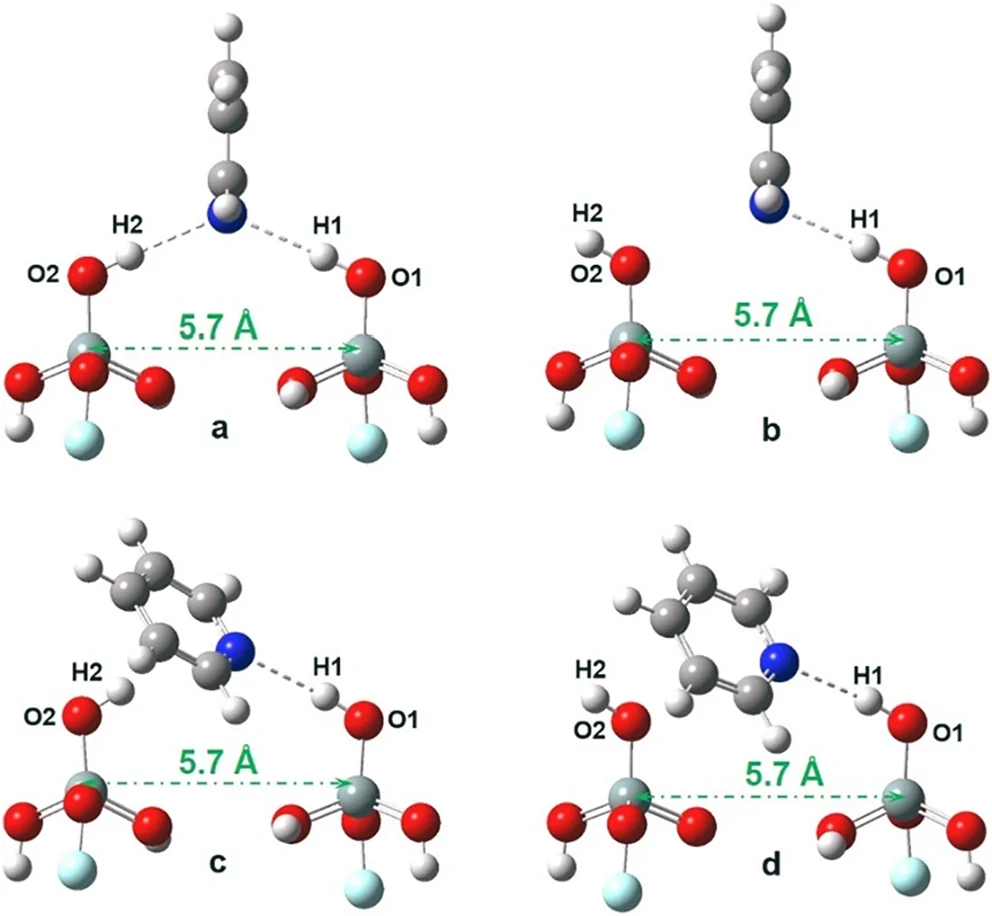
A model pattern of surface silanol groups of MCM-41 with adsorbed pyridine. Only the positions of the H1, H2, O1, and O2 atoms were optimized. Sil^in^_Py_tr (a), Sil^out^_Py_tr (b), Sil^in^_Py_pretr (c), Sil^out^_Py_pretr (d).

Although the hydrogen bonds in Sil^in^_Py_tr are long, H···N distances of 2.20 Å, and nonlinear, with angles of about 114° between the O–H bond directions and the C_2_ symmetry axis, the energy of each bond is approximately 16.5 kJ/mol. When one of these bonds is removed, Sil^out^_Py_tr, the total energy increases to 36.1 kJ/mol, while the remaining bond becomes only slightly stronger, Figure 2b and Table 1.

The N···O distances in Sil^in^_Py_tr are 3.172 Å. An intermediate structure between Sil^in^_Py_tr and Sil^in^_Py is expected to be characterized by pyridine forming a linear hydrogen bond with one of the silanol groups, with the corresponding N···O distance elongated to 3.172 Å. This structure is denoted as Sil^in^_Py_pretr, Figure 2c. The energy of Sil^in^_Py_pretr is 13.3 kJ/mol higher than that of Sil^in^_Py and 6.6 kJ/mol lower than that of Sil^in^_Py_tr, Table 1. In this structure, the interaction of pyridine with the second silanol group is stronger than in Sil^out^_Py, which facilitates the transition to Sil^in^_Py_tr.

## Conclusions

The intuitive expectation that a distance of about 6 Å between neighboring silanol groups, expected for highly ordered MCM-41, would be sufficient to prevent an adsorbed molecule with a single proton-accepting center from forming multiple hydrogen bonds is not valid. In fact, pyridine molecules adsorbed on a surface with a shortest silanol–silanol distance of as much as 5.7 Å can form two bifurcated hydrogen bonds, each with an energy of approximately 16.5 kJ/mol.

As a result, the activation barrier for unrestricted, smooth surface diffusion of adsorbed pyridine is only about 20 kJ/mol and can be readily overcome at room temperature, in contrast to the desorption barrier, which exceeds 50 kJ/mol. The N···O distances in these bifurcated hydrogen bonds are about 3.17 Å, compared to 2.68 Å in the most energetically favorable structure featuring a single dominant hydrogen bond. Elongation of this bond to 3.17 Å requires approximately 13 kJ/mol, while an additional 7 kJ/mol is required to disrupt its linearity and reorient the pyridine molecule to form two equivalent nonlinear hydrogen bonds.

The mean density of surface silanol groups in disordered amorphous silica is approximately 5 OH/nm^2^, compared to about 3 OH/nm^2^ for ordered MCM-41. Accordingly, the shortest silanol–silanol distance in such disordered materials is necessarily smaller than 5.7 Å and likely does not exceed 4.5 Å. As a result, the associated activation barrier for surface diffusion is expected to be significantly lower than 20 kJ/mol, whereas desorption still requires more than 50 kJ/mol. These findings indicate that bifurcated hydrogen bonds constitute a key mechanism governing the mobility of adsorbed molecules on silica surfaces, provided that such molecules possess an accessible proton-accepting center, and thus play an integral role in heterogeneous chemical processes.

## Supplementary Material



## Notes

Molecular structures of Sil^in^_Py (Table S1); Sil^out^_Py (Table S2); Sil^in^_Py_tr (Table S3); Sil^out^_Py_tr (Table S4); Sil^in^_Py_pretr (Table S5); and Sil^out^_Py_pretr (Table S6) (PDF)
