# Supplementary material for: Hydrogen-Bond Bifurcation as the Key Mechanism of Pyridine Surface Diffusion on MCM-41
Source: ACS Omega. 2026 Sep 4;11(37):55437–41. doi: 10.1021/acsomega.6c04133 (PMC13613477; doi:10.1021/acsomega.6c04133)
Supplement: Supplementary file 1 [file ao6c04133_si_001.pdf]

Supporting information for

Hydrogen-Bond Bifurcation as the Key Mechanism of Pyridine Surface Diffusion on MCM-41

Ilya G. Shenderovich[*]

Faculty of Chemistry and Pharmacy, University of Regensburg, Universitätsstr. 31, 93040, Regensburg, Germany

Content Page

Table S1	The molecular structure of Sil^{in}_Py	S2
Table S2	The molecular structure of Sil^{out}_Py	S3
Table S3	The molecular structure of Sil^{in}_Py_tr	S4
Table S4	The molecular structure of Sil^{out}_Py_tr	S5
Table S5	The molecular structure of Sil^{in}_Py_pretr	S6
Table S6	The molecular structure of Sil^{out}_Py_pretr	S7

Table S1. The molecular structure of Sil^{in}_Py.

Si,-1,2.8499129806,-0.0000164746,0.0000226296
O,0,2.9941091226,-1.6043666124,-0.0200782112
H,0,2.259342455,-2.1579278905,0.2531843316
O,-1,2.8499890574,0.3700521626,1.6595596333
O,-1,1.4765549221,0.6436389275,-0.5743523856
O,-1,4.1860836538,0.5801722194,-0.7208073916
F,-1,2.9405257385,1.7892737879,1.7466282694
H,-1,1.4119825186,0.8135385719,-1.5146500028
H,-1,4.2885519677,1.5274194823,-0.8195712086
Si,-1,-2.8499134996,-0.0000141864,-0.0000175593
O,0,-2.9443535701,-1.5951893858,-0.0175759441
H,0,-2.1182289241,-2.1412094885,0.1379664629
O,-1,-2.849988911,0.3700539495,-1.6595592348
O,-1,-1.4765484295,0.6436301646,0.5743469342
O,-1,-4.1860871157,0.5801705459,0.7208023221
F,-1,-2.9405285983,1.7892736027,-1.7466283106
H,-1,-1.4119855521,0.8135460639,1.5146488588
H,-1,-4.2885487325,1.5274191827,0.8195774459
N,0,-0.7508586619,-3.0512682105,0.4667055826
C,0,1.4997993446,-4.5335643842,1.0992537793
C,0,-0.2985434307,-3.9886207816,-0.3639261091
C,0,-0.0974348269,-2.8401422652,1.6082271773
C,0,0.8228109979,-4.7531387936,-0.0899549414
C,0,1.0328725526,-3.5558155487,1.966274402
H,0,-0.8566647003,-4.1276985858,-1.2819749161
H,0,1.1546586128,-5.50164358,-0.795451767
H,0,2.3809710478,-5.1109283329,1.346049707
H,0,1.5326142877,-3.3484765339,2.9020239605
H,0,-0.4947357911,-2.0667090958,2.2547835431

Table S2. The molecular structure of Sil^{out}_Py.

Si,-1,2.849894,-0.000021,0.000031
O,0,2.8239428157,-1.6083386104,-0.0243211837
H,0,3.5963503024,-2.1096428989,0.2391057606
O,-1,2.849982,0.370056,1.659559
O,-1,1.476556,0.64364,-0.574354
O,-1,4.186084,0.580167,-0.72081
F,-1,2.940533,1.789273,1.746628
H,-1,1.411984,0.813542,-1.51465
H,-1,4.288551,1.527419,-0.819572
Si,-1,-2.849892,-0.000014,-0.000037
O,0,-2.9248692927,-1.5964300127,-0.0170635053
H,0,-2.1006103613,-2.1216760436,0.2191472818
O,-1,-2.84999,0.370055,-1.659559
O,-1,-1.476563,0.643659,0.574349
O,-1,-4.186071,0.580162,0.720839
F,-1,-2.940531,1.789273,-1.746628
H,-1,-1.411977,0.813525,1.514652
H,-1,-4.288562,1.52742,0.819551
N,0,-0.7522728006,-2.9937964244,0.6848201016
C,0,1.4118629408,-4.4775326286,1.5662035842
C,0,-0.2668303926,-3.9925247696,-0.0505911328
C,0,-0.169802594,-2.7229182116,1.8517297978
C,0,0.8123284563,-4.7605318356,0.3497614675
C,0,0.9125394342,-3.4375240742,2.3327392228
H,0,-0.7659589984,-4.1812933959,-0.9937318195
H,0,1.1704229076,-5.5618064937,-0.2814493006
H,0,2.2577301858,-5.0582235184,1.9105399326
H,0,1.3515572338,-3.1794882936,3.2863206402
H,0,-0.5915553339,-1.9022259356,2.4200293955

Table S3. The molecular structure of Sil^{in}_Py_tr.

Si,-1,2.85002714,0.0000086391,-0.0000067901
O,0,2.8858154922,-1.6120387867,0.0033487915
H,0,2.0158702011,-2.0363404122,-0.0076001115
O,-1,2.8500018446,0.3700570169,1.6595585503
O,-1,1.476491131,0.6435147359,-0.5743393013
O,-1,4.1860993083,0.580182328,-0.7207702374
F,-1,2.9405013688,1.7892753641,1.7466279188
H,-1,1.4120155586,0.813620216,-1.5146375161
H,-1,4.2885338185,1.5274177969,-0.8196062073
Si,-1,-2.8500271402,0.0000086394,0.0000067913
O,0,-2.8858152995,-1.6120387895,-0.0033486358
H,0,-2.0158699544,-2.0363402426,0.0076031489
O,-1,-2.8500018444,0.3700570168,-1.6595585503
O,-1,-1.4764911306,0.6435147351,0.5743393012
O,-1,-4.1860993081,0.580182328,0.7207702378
F,-1,-2.9405013688,1.7892753641,-1.7466279188
H,-1,-1.4120155588,0.8136202166,1.514637516
H,-1,-4.2885338188,1.5274177969,0.8196062067
N,-1,-0.0000000001,-2.927922733,0.0000059991
C,-1,0.,-5.713221,0.
C,-1,0.0000025722,-3.6221182304,-1.137924979
C,-1,-0.0000025721,-3.6221182301,1.1379249791
C,-1,-0.0000009759,-5.005717,-1.19112
C,-1,0.000000976,-5.005717,1.19112
H,-1,0.0000024187,-3.03954,-2.054375
H,-1,0.,-5.51289,-2.14646
H,-1,0.,-6.795726,0.
H,-1,0.,-5.51289,2.14646
H,-1,-0.0000024188,-3.03954,2.054375

Table S4. The molecular structure of Sil^{out}_Py_tr.

Si,-1,2.8500238318,0.00001007,0.000030085
O,0,2.8751221834,-1.612246055,0.0039191503
H,0,1.9997381684,-2.0290980064,-0.0075335982
O,-1,2.8500053123,0.3700600788,1.6595583135
O,-1,1.4764912156,0.6435135027,-0.5743357579
O,-1,4.1861004755,0.5801809295,-0.7207681266
F,-1,2.9405017084,1.7892749542,1.7466280102
H,-1,1.4120141782,0.8136154956,-1.51463869
H,-1,4.2885314988,1.5274177672,-0.8196108239
Si,-1,-2.8500384088,0.000025625,-0.0000367238
O,0,-2.8784639422,-1.609102051,0.0068669578
H,0,-3.7157318253,-2.0549862811,-0.1290214572
O,-1,-2.8500589711,0.3700480002,-1.6595610076
O,-1,-1.4764611692,0.6435169383,0.5742695604
O,-1,-4.1860700054,0.5801878969,0.7208190004
F,-1,-2.9405036861,1.7892748263,-1.7466280388
H,-1,-1.4120183804,0.8136258854,1.5146370985
H,-1,-4.2885284859,1.5274174867,0.8196166347
N,-1,0.0000025449,-2.9279397588,0.0000691477
C,-1,0.,-5.713221,0.
C,-1,0.0000189884,-3.6220833669,-1.1379461279
C,-1,-0.0000130122,-3.6221533312,1.137903446
C,-1,0.0000106098,-5.005717,-1.1911199999
C,-1,0.0000075741,-5.005717,1.19112
H,-1,0.0000025359,-3.03954,-2.054375
H,-1,0.,-5.51289,-2.14646
H,-1,0.,-6.795726,0.
H,-1,0.,-5.51289,2.14646
H,-1,-0.0000183545,-3.03954,2.054375

Table S5. The molecular structure of Sil^{in}_Py_pretr.

Si,-1,2.850059475,-0.000000563,-0.0000050061
O,0,2.9303884611,-1.6067432685,0.0200080821
H,0,2.1182155283,-2.0820871044,-0.2389085813
O,-1,2.850001441,0.3700585408,1.6595586564
O,-1,1.4764900547,0.6435137407,-0.5743381073
O,-1,4.1861086452,0.5801906354,-0.7207451688
F,-1,2.9404952001,1.7892753749,1.7466279162
H,-1,1.4120159445,0.8136198629,-1.514638021
H,-1,4.2885217633,1.5274168586,-0.8196296024
Si,-1,-2.8500570979,0.0000022094,0.0000056716
O,0,-2.9728297136,-1.6064591244,0.0090380928
H,0,-2.1764985084,-2.1416692616,0.0051459031
O,-1,-2.8500093124,0.3700563418,-1.659559147
O,-1,-1.4764974781,0.6435374414,0.5743290919
O,-1,-4.1860986681,0.5801882562,0.7207655791
F,-1,-2.9404926371,1.7892755405,-1.7466278792
H,-1,-1.4120079886,0.8136001943,1.5146410324
H,-1,-4.2885293417,1.5274175661,0.8196149842
N,0,0.2483628657,-2.9274123154,-1.0392315734
C,0,-1.9340447898,-4.0451168445,-2.3428075851
C,0,-0.196992182,-2.4231055542,-2.1890217339
C,0,-0.3829525668,-3.9876510058,-0.5338833875
C,0,-1.2811316205,-2.9460318049,-2.8754154225
C,0,-1.4773030178,-4.5772024992,-1.1468443316
H,0,0.3363116739,-1.562565303,-2.5755647336
H,0,-1.6038519828,-2.4948754912,-3.8033867398
H,0,-2.7871090963,-4.4784122773,-2.8482381371
H,0,-1.9570938048,-5.4327168688,-0.6920735088
H,0,0.0027483905,-4.3773293902,0.4010080369

Table S6. The molecular structure of Sil^{out}_Py_pretr.

Si,-1,-3.181969,0.233844,-0.179896
O,0,-2.8255830649,1.6881558262,0.4076002546
H,0,-1.8826971368,1.9438005206,0.4031579674
O,-1,-3.642551,-0.605138,1.225475
O,-1,-1.945283,-0.618473,-0.792124
O,-1,-4.43627,0.378293,-1.203116
F,-1,-4.133084,-1.868829,0.786831
H,-1,-1.727366,-0.503449,-1.717618
H,-1,-4.769016,-0.403131,-1.646016
Si,-1,2.161296,-1.649714,0.447046
O,0,2.5688757227,-0.2171550235,1.0536083858
H,0,3.4713095516,0.0993007829,1.00101738
O,-1,2.420158,-1.471421,-1.22397
O,-1,0.573815,-1.946322,0.597312
O,-1,3.099307,-2.830014,1.053795
F,-1,2.137022,-2.741546,-1.804113
H,-1,0.263163,-2.365008,1.40072
H,-1,2.915714,-3.739731,0.816696
N,0,0.2553884558,2.3936224035,0.1442533559
C,0,2.9144510917,2.9767954558,-0.4022989968
C,0,0.8123957654,2.0414352361,-1.0125833121
C,0,1.014632337,3.0301623709,1.033402052
C,0,2.132867444,2.3103369296,-1.3313645434
C,0,2.344826428,3.3429959402,0.8062005426
H,0,0.1722032927,1.5222251001,-1.7165146659
H,0,2.5341413745,2.0004451825,-2.2864648215
H,0,3.9509398822,3.2049178185,-0.6152721812
H,0,2.9163500413,3.8627723029,1.5629195287
H,0,0.5366836616,3.3027258943,1.9675584384
